# Case report: Transcatheter pulmonary valve-in-valve implantation in a deteriorated self-expandable valve caused by infective endocarditis

**DOI:** 10.3389/fcvm.2022.939297

**Published:** 2022-08-24

**Authors:** Yan-Jie Li, Xin Pan, Cheng Wang, Ben He

**Affiliations:** Department of Cardiology, Shanghai Chest Hospital, Shanghai Jiao Tong University, Shanghai, China

**Keywords:** degenerated bioprosthesis, infective endocarditis, tetralogy of Fallot, transcatheter pulmonary valve implantation, valve-in-valve (VIV)

## Abstract

**Background:**

Infective endocarditis is a complication with high mortality in patients with congenital heart disease, particularly for those with bioprosthetic valve.

**Case summary:**

We report a case of a 54-year-old female with a history of tetralogy of Fallot who had been surgically repaired using a transannular patch due to severe pulmonary insufficiency with right heart enlargement and presented with worsening dyspnea. She had received transcatheter pulmonary valve implantation (TPVI) 5 years ago. Unfortunately, bioprosthesis-associated infective endocarditis occurred due to dental caries. Given persistent antibiotic medication, she became clinically stable with prosthesis functional recovery. However, dysfunctional bioprosthesis was still detected 3 years later, which was successfully treated by valve-in-valve TPVI with the help of modified buddy wire technique. At a 12-month follow-up after valve-in-valve TPVI, she was completely recovered with improved symptoms of heart failure.

**Conclusion:**

This is the first report of valve-in-valve TPVI of a self-expandable valve in a degenerated self-expandable valve. The case highlights increased surveillance for infective endocarditis of transcatheter pulmonary valve should be emphasized. Subsequent valve-in-valve TPVI is an effective treatment for valve failure in defined conditions improving the hemodynamics.

## Introduction

Pulmonary regurgitation is a common late consequence of surgical repair of tetralogy of Fallot (TOF) (1, 2). European society of cardiology guidelines on the management of adult congenital heart disease recommend transcatheter pulmonary valve implantation (TPVI) in symptomatic patients affected by severe pulmonary regurgitation (3). Given the relatively low risk of procedural complications, TPVI may be an optimal option in selective cases. Although TPVI has been reported to be a feasible procedure with successful implantation rate >95% (4, 5), it remains challenging and technically demanding in some special scenario.

In this report, we present a treatment modality for self-expandable valve failure caused by infective endocarditis (IE), using valve-in-valve TPVI with the assistance of modified buddy wire technique.

## Case description

**Figure 1:**
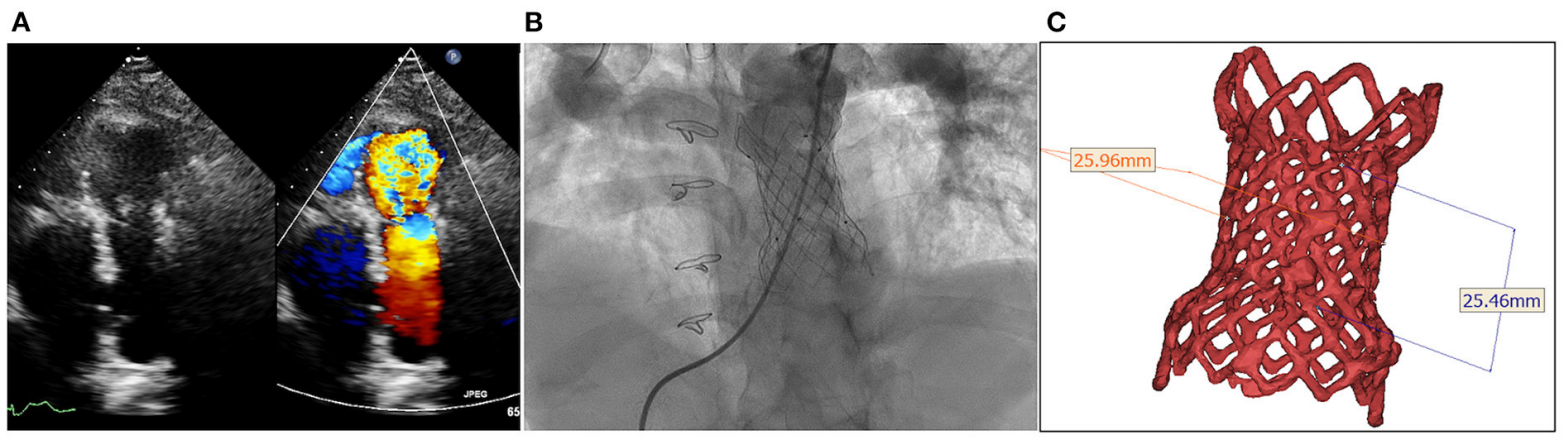
Degenerated bioprosthesis with complete frame. **(A)** Transthoracic echocardiography shows severe pulmonary regurgitation; **(B)** Angiography reveals severe pulmonary insufficiency; **(C)** The annular size calculated on computed tomography.

A 54-year-old female with a history of TOF was admitted to our hospital with complains of exertional dyspnea and chest distress. She underwent surgery for TOF with transannular patching of right ventricular outflow tract (RVOT) at the age of 18. Five years ago, she received TPVI, using a P26-25 mm (diameter-length) self-expandable Venus P-valve (Venus Medtech, Hangzhou, China) due to severe pulmonary regurgitation with right heart enlargement. Unfortunately, bioprosthesis-associated IE occurred due to dental caries 6 months after TPVI. Transthoracic echocardiography (TTE) showed vegetation attached on the prosthetic valve and blood culture indicated streptococcus viridians. At the end of 6 weeks of intravenous penicillin and gentamycin, her blood cultures sterilized, her vegetation size was reduced, and she achieved prosthesis functional recovery (6). Then, she received dental caries extraction under antibiotic prophylaxis with intravenous penicillin 80 million units/day for 3 days. During close follow-up, she had not got relapse of IE. However, dysfunctional bioprosthesis was still detected by TTE with thicken leaflet, severe pulmonary regurgitation 3 years later (Figure 1A; Supplementary Video 1), moderate to severe tricuspid insufficiency and right heart dilation. Considering the relatively low risk of procedural complications, a decision of valve-in-valve TPVI with a self-expandable valve was made.

**Figure 2:**
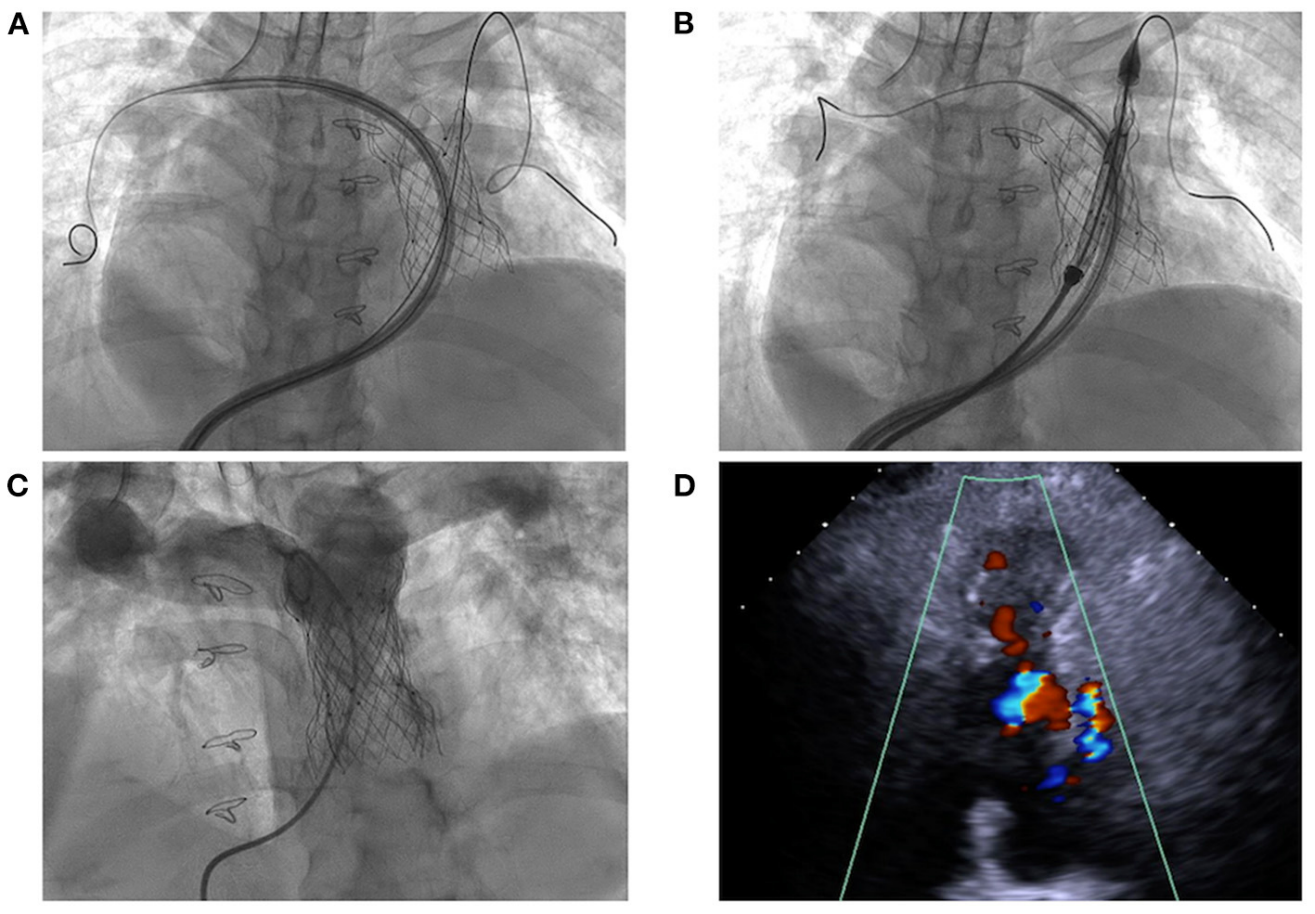
Transcatheter pulmonary valve-in-valve implantation for degenerated bioprosthesis. **(A,B)** The delivery system crossed the degenerated bioprostheses with the assistance of modified buddy wire; **(C,D)** The result of transcatheter valve-in-valve implantation with no pulmonary regurgitation.

**Figure 3:**
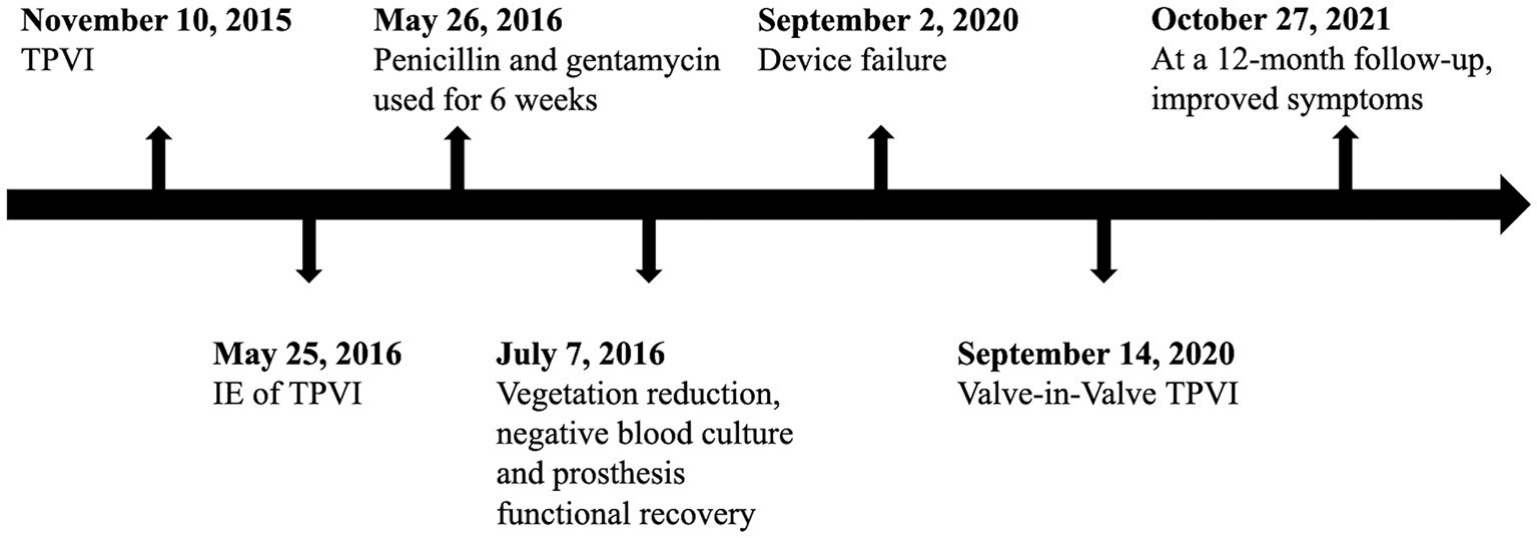
Timeline of events of the patient.

The procedure was performed with fluoroscopic and TTE guidance under general anesthesia. Bilateral femoral veins were used, and right heart catheterization was first executed. Pulmonary artery pressure was 35/8 mmHg and right ventricle pressure was 50/8 mmHg. Severe pulmonary insufficiency appeared on angiography (Figure 1B; Supplementary Video 2). After pre-dilation with a 25 mm × 50 mm (diameter × length) balloon (Balt, Montmorency, France), systolic pressure gradient across the bioprosthesis decreased from 15 mmHg to 6 mmHg. Since the annular size of 25.9 mm calculated on computed tomography and three-dimension reconstruction (Figure 1C), a P28-25 mm (diameter-length) self-expandable Venus P-valve (Venus Medtech, Hangzhou, China) with 22-F delivery system was planned to be deployed. The process was beset with difficulties in advancing the delivery sheath. The buddy wire technique, which was employed with two Lunderquist wires, was attempted but the delivery system still failed to be advanced and positioned appropriately. Finally, the 14-F Cook sheath (Cook Medical, Bloomington, USA) was advanced over the second Lunderquist wire, which passed through the RVOT and placed in the distal of right pulmonary artery. The 14-F Cook sheath served as modified buddy wire providing extra support and straightening the vessel and the RVOT (Figure 2A). Maneuvering the 22-F Venus P delivery system alongside the 14-F Cook sheath aided in the advancement of the delivery system into the proximal end of left pulmonary. Prior to the deployment of the second pulmonary valve, the 14-F Cook sheath was pulled back into inferior vena cava. The new self-expanding valve was then successfully delivered (Figure 2B) and implanted. Post-dilation with a 25 mm × 50 mm (diameter × length) balloon (Balt, Montmorency, France) was performed to better shape for the stent strut and RVOT. Post-procedure angiography and TTE showed no pulmonary regurgitation (Figures 2C,D, 3; Supplementary Videos 3, 4). Then pulmonary pressure was raised to 35/18 mmHg.

During hospitalization, the patient was given adequate and prolonged antibiotic prophylaxis (intravenous ceftriaxone 2 g/day and vancomycin 1 g/day for 1 week) to ensure no endocarditis relapse. She was recommended to take oral anticoagulant daily after discharge and reinforced follow-up was proposed to her as before. At 12-month follow-up, she was completely recovered from valve-in-valve TPVI with improved symptoms of heart failure (Figure 3). TTE was obtained and showed a persistently good valve function.

## Discussion

TPVI is an effective treatment alternative to surgical pulmonary valve replacement for patients with right ventricular dysfunction after correction of TOF (7–10). IE of TPVI is a dreadful complication and associated with a relevant need of re-intervention (11). IE of Venus P valve with concomitant COVID-19 infection was also reported (12). In our case, the mechanism resulting in recurrence of pulmonary regurgitation after TPVI was IE, eventually leading to recurrence of pulmonary regurgitation and right heart failure. Although transcatheter procedure could be the first reintervention, it was necessary to identify that IE was adequately treated, which was confirmed by no clinical sign and no vegetations on echocardiogram in this scenario.

This is the first report of valve-in-valve TPVI in a degenerated self-expandable pulmonary valve. Maneuvering the relatively rigid delivery system can be challenging in a dilated and tortuous right ventricle with severe tricuspid insufficiency, and it is difficult to advance the valve especially in a stented RVOT. Although several reported technical and therapeutic considerations remain to be resolved in difficult TPVI cases (13–15), the modified buddy wire technique including super stiff wire combined with long sheath to provide extra guide and support could be a bailout approach, which facilitates to maneuver the delivery system crossing the basal valve frame and deploy the new valve at accurate position. Albeit limited data were available regarding the hemodynamic efficacy and durability of such intervention, repeat TPVI was still an effective treatment for valve failure in defined conditions, improving by freedom from re-intervention, which further adds to the benefit of TPVI in the life-time management of RVOT dysfunction (16). During the procedure, the technique along with the wire and sheath can be used to place percutaneous valve in accurate position.

In conclusion, this case highlights reinforced surveillance for IE of transcatheter pulmonary valve should be emphasized. Subsequent valve-in-valve TPVR is an effective treatment for early bioprosthetic failure in defined conditions. Repeat TPVI is particularly challenging in case with self-expandable valve but is an effective treatment with the modified buddy wire technique to provide extra support for delivery system.

## Data availability statement

The original contributions presented in the study are included in the article/Supplementary material, further inquiries can be directed to the corresponding author.

## Ethics statement

The studies involving human participants were reviewed and approved by the Human Ethics Committee of Shanghai Chest Hospital, Shanghai Jiao Tong University. The patients/participants provided their written informed consent to participate in this study. Written informed consent was obtained from the individual(s) for the publication of any potentially identifiable images or data included in this article.

## Author contributions

Y-JL, XP, CW, and BH contributed to the conception, design, and implement of the study. Y-JL and XP organized the data, performed the statistical analysis, and wrote the manuscript. All authors contributed to manuscript, read, and approved the submitted version for publication.

## Funding

This study was supported by grants from the Shanghai Committee of Science and Technology (17411970900 and 22YF1443000) and Clinical Research Plan of SHDC (SHDC2020CR1039B), China.

## Conflict of interest

The authors declare that the research was conducted in the absence of any commercial or financial relationships that could be construed as a potential conflict of interest.

## Publisher's note

All claims expressed in this article are solely those of the authors and do not necessarily represent those of their affiliated organizations, or those of the publisher, the editors and the reviewers. Any product that may be evaluated in this article, or claim that may be made by its manufacturer, is not guaranteed or endorsed by the publisher.

## Supplementary material

Supplementary video S1 Thickened leaflet and severe pulmonary regurgitation on echocardiography.

Supplementary video S2 Severe pulmonary regurgitation on angiography.

Supplementary video S3 Post-procedure angiography showed no pulmonary regurgitation.

Supplementary video S4 No regurgitation after transcatheter valve-in-valve implantation on echocardiography.

## References

[1] Greutmann M Ruperti J Schwitz F Haag N Santos Lopes B Meier L. High variability of right ventricular volumes and function in adults with severe pulmonary regurgitation late after tetralogy of fallot repair. Am J Cardiol. (2022) 166:88–96. 10.1016/j.amjcard.2021.11.022 34949470

[2] Liu J Jiang X Peng B Li S Yan J Wang Q. Association of pulmonary valve morphology differences with outcomes in tetralogy of fallot repair with right ventricular outflow tract incision. Front Cardiovasc Med. (2021) 8:695876. 10.3389/fcvm.2021.695876 34422925 PMC8372408

[3] Baumgartner H De Backer J Babu-Narayan SV Budts W Chessa M Diller GP. 2020 ESC Guidelines for the management of adult congenital heart disease. Eur Heart J. (2021) 42:563–645. 10.15829/1560-4071-2021-4702 32860028

[4] Sinha S Aboulhosn J Levi DS. Transcatheter pulmonary valve replacement in congenital heart disease. Interv Cardiol Clin. (2019) 8:59–71. 10.1016/j.iccl.2018.08.006 30449422

[5] Tannous P Nugent A. Transcatheter pulmonary valve replacement in native and nonconduit right ventricle outflow tracts. J Thorac Cardiovasc Surg. (2021) 162:967–70. 10.1016/j.jtcvs.2020.07.126 33097216

[6] Wang C Li YJ Ma L Pan X. Infective endocarditis in a patient with transcatheter pulmonary valve implantation. Int Heart J. (2019) 60:983–5. 10.1536/ihj.18-497 31257331

[7] Jones TK McElhinney DB Vincent JA Hellenbrand WE Cheatham JP Berman DP. Long-term outcomes after Melody transcatheter pulmonary valve replacement in the US investigational device exemption trial. Circ Cardiovasc Interv. (2022) 15:e010852. 10.1161/CIRCINTERVENTIONS.121.010852 34930015 PMC8765216

[8] Lee SY Kim GB Kim SH Jang SI Choi JY Kang IS. Mid-term outcomes of the Pulsta transcatheter pulmonary valve for the native right ventricular outflow tract. Catheter Cardiovasc Interv. (2021) 98:E724–32. 10.1002/ccd.29865 34227733

[9] McElhinney DB Zhang Y Levi DS Georgiev S Biernacka EK Goldstein BH. Reintervention and survival after transcatheter pulmonary valve replacement. J Am Coll Cardiol. (2022) 79:18–32. 10.1016/j.jacc.2021.10.031 34991785

[10] Morgan G Prachasilchai P Promphan W Rosenthal E Sivakumar K Kappanayil M. Medium-term results of percutaneous pulmonary valve implantation using the Venus P-valve: international experience. EuroIntervention. (2019) 14:1363–70. 10.4244/EIJ-D-18-00299 30248020

[11] Bos D De Wolf D Cools B Eyskens B Hubrechts J Boshoff D. Infective endocarditis in patients after percutaneous pulmonary valve implantation with the stent-mounted bovine jugular vein valve: clinical experience and evaluation of the modified Duke criteria. Int J Cardiol. (2021) 323:40–6. 10.1016/j.ijcard.2020.08.058 32860844

[12] Chimoriya R Awasthy N Kumar G. COVID-19 infection with delayed presentation of infective endocarditis of the prosthetic pulmonary valve. Cardiol Young. (2021) 31:2045–7. 10.1017/S1047951121002080 34092265

[13] Baz Alonso JA Fernandez GB Barbeira SF Romo AI Zunzunegui JL Jimenez-Diaz VA. Transcatheter pulmonary valve replacement with “Double-Barrel” stent-and-valve technique in a dilated right ventricular outflow tract. JACC Cardiovasc Interv. (2021) 14:e283–e4. 10.1016/j.jcin.2021.08.015 34600878

[14] Rudzinski PN Kalinczuk L Mintz GS Demkow M. Large field-of-view intravascular ultrasound offering tomographic perspective online for accurate sizing during transcatheter pulmonary valve replacement. Eur Heart J Cardiovasc Imaging. (2022) jeac075. 10.1093/ehjci/jeac075 PMC9757892 35511055

[15] Shah RR Poommipanit P Law MA Amin Z. Anchor balloon, buddy wire, and wire and sheath techniques to deploy percutaneous pulmonary valves in tetralogy of fallot patients. Catheter Cardiovasc Interv. (2018) 92:915–20. 10.1002/ccd.27022 28303658

[16] Nordmeyer J Coats L Lurz P Lee TY Derrick G Rees P. Percutaneous pulmonary valve-in-valve implantation: a successful treatment concept for early device failure. Eur Heart J. (2008) 29:810–5. 10.1093/eurheartj/ehn073 18316357

